# Prompt Mental Health Care (PMHC): work participation and functional status at 12 months post-treatment

**DOI:** 10.1186/s12913-020-4932-1

**Published:** 2020-02-04

**Authors:** Marit Knapstad, Solbjørg Makalani Myrtveit Sæther, Gunnel Hensing, Otto Robert Frans Smith

**Affiliations:** 1 Department of Clinical Psychology, University of Bergen, P.B. 7807, N-5020 Bergen, Norway; 2 Department of Health Promotion, Norwegian Institute of Public Health, Zander Kaaes gate 7, N-5015 Bergen, Norway; 3 School of Public Health and Community Medicine, Institute of Medicine at Sahlgrenska Academy, University of Gothenburg, Gothenburg, Sweden

**Keywords:** Anxiety, Depression, Early intervention, Public mental health, Treatment

## Abstract

**Background:**

Anxiety and depression are associated with substantial functional impairment. Prompt Mental Health Care (PMHC), the Norwegian adaptation of IAPT is currently piloted across Norway, as a means to improve access to evidence-based care for adults with anxiety disorders (including subthreshold cases) and minor to moderate depression. The aims of the current paper were to examine the change in work status and functional status from pre- to post-treatment and 12 months post-treatment among clients at the first 12 PMHC pilot sites, and whether degree of change differed across sociodemographic characteristics.

**Methods:**

A prospective cohort design was used, including working age clients receiving treatment between October 2014 and December 2016 (*n* = 1446, participation rate = 61%). Work status and functional status were self-reported, the latter by the Work and Social Adjustment Scale (WSAS). Changes in work status and WSAS score were examined through multilevel models based on maximum likelihood estimation. Likelihood ratio tests were performed to determine whether the interaction between time and the respective background variables were statistically significant.

**Results:**

A substantial increase in regular work participation was observed from pre- to post-treatment, which further had increased at 12 months post-treatment. The increase was driven by a corresponding reduction in proportion of clients working and receiving benefits (OR 0.38 [0.29–0.50] baseline to final treatment, OR = 0.19 [0.12–0.32] final treatment to 12-months post-treatment), while no statistically significant change was observed in proportion out of work. Large improvement (ES = − 0.89) in WSAS score was observed from pre- to post treatment. WSAS score at 12 months post-treatment remained at the post-treatment level.

**Conclusions:**

Previous research has shown substantial symptom improvement among clients receiving treatment in PMHC. The current findings indicate that PMHC might also be able to aid adults struggling with mild to moderate anxiety and depression in returning to usual level of functioning. The degree to which the observed improvements are attributable to the treatment need nonetheless to be confirmed in a trial including a control group and with more complete follow-up data from registries.

## Background

Anxiety and depression are associated with substantial functional impairment, impacting both private life and the occupational sphere in terms of presenteeism and absenteeism [1–6]. The functional impairment is greater for these than many other common conditions, such as back pain, diabetes and arthritis [7, 8]. Further, function may also be impaired among workers with subthreshold symptom levels [9, 10] and after remission [5, 11, 12]. From an individual point of view, it is therefore not surprising that return to usual level of functioning is considered by depressed individuals themselves to be among the key facets defining remission from depression [13]. From a societal point of view, the functional impairment in combination with the high point prevalence of anxiety (7%) [14] and depression (4%) [15], and typical early adulthood onset [16], make these conditions immensely costly. Anxiety and depression constitute two of the major causes of burden of disease [17] and temporary and permanent work-life absence among working-age adults [18–20]. Notably, though there is no clear evidence for a general increase in prevalence of mental disorders, reports across several Western countries show that their impact on work absence is ever increasing [21–23].

While anxiety and depression are important causes for work absence, there is strong evidence for work to have a protective effect on mental health [24] and that unemployment and sick leave itself can be detrimental for health [25, 26]. Work gives access to monetary resources, and may provide a daily structure, identity and meaningful activities [27]. The workplace is also an important arena for social inclusion for working-age adults in modern society.

Finding measures to lower the burden of disease from anxiety and depression and facilitate return to work and work retention among people struggling with anxiety and depression are therefore highly prioritized tasks across many countries. Two major challenges need to be solved in order to meet these goals. Firstly, there is a huge gap between the numbers suffering from anxiety and depression and the numbers seeking and receiving minimal adequate treatment, both in Norway [28] and globally [2, 29, 30]. Secondly, while there are substantial evidence that psychological treatments, such as cognitive behavioural therapy (CBT), have good effect on symptom reduction and wellbeing [31, 32] the degree to which such interventions have effect on functional and work outcomes remain unsettled [33, 34]. There is notably growing evidence that incorporating an explicit work-focus in CBT treatments has a better effect on work outcomes than CBT alone [32, 35, 36]. The picture still is not clear-cut [37, 38] and few studies have included longer-term follow-up [36].

Prompt Mental Health Care (PMHC, “Rask psykisk helsehjelp” in Norwegian) was initiated by the Norwegian Directorate of Health (NDH), as a means to improve access to evidence-based care for adults with anxiety disorders and subthreshold to moderate depression. PMHC is based on the English, innovative program “Improving access to psychological therapies” (IAPT) [39]. In short, PMHC offers low-threshold, free-of-charge CBT-based treatment. The treatment is organized according to a matched care model including both low (guided self-help, group-based psychoeducation) and high (individual CBT) intensity treatment types [40]. Data from the 12 first PMHC pilot sites have shown promising results in terms of large improvements (effect sizes 1.0–1.2) in symptoms of anxiety and depression from pre- to post treatment [41]. The observed improvement was also largely maintained at 12 months post-treatment [42].

Increased work participation is, second to alleviation of symptoms, a key objective for the PMHC and IAPT programs. In fact, gains achieved by reducing presenteeism and absenteeism were of the chief arguments used for the cost-effectiveness of a large-scale roll-out of the IAPT program [43, 44]. Several of the features of PMHC and IAPT align well with the current evidence-base for interventions to facilitate return to work and work participation for people who struggle with anxiety and depression. Firstly, early intervention might be decisive in preventing long-term or permanent work-life exclusion. For instance, time before beginning psychotherapy is found to predict the length of sick leave when the variables age and duration of psychotherapy were controlled [45]. Secondly, and reassuring for treatment models including low-intensity care such as PMHC, a Cochrane review found moderate quality evidence based on three studies for telephone or online CBT to be more effective in reducing sick leave than usual primary or occupational care among depressed people [35]. A stepped, primary care intervention for depression has also shown small to moderate functional improvements at up to 3 months follow-up [46]. Nevertheless, as stated by Thornicroft (2018), there still is little direct evidence that the IAPT program in facts meet the goal of reducing presenteeism and absenteeism [47].

When evaluating functional outcomes of an intervention work status in combination with perceived functional status can give crucial and complimentary information. While work status is an important indicator of economic independency and societal costs, this is only one aspect of functioning. Perceived functional status gives information about the subjective experience of daily life functioning in a broader sense, both privately and at work. Also, affected function is a criterion for a formal mental disorder diagnosis. Such measures can therefore be considered particularly informative, in addition to symptomatology, when evaluating low-threshold interventions where no formal diagnosis is set, as in PMHC. Finally, considering the many structural factors impacting on work status, such as labour market situation and benefit systems, but also cultural factors like how mental disorders are perceived and treated at workplaces [48], it is not given that the two measures follow the same recovery path. For instance, in the current focus on early return to work in Norway, a client may have returned to work despite still experiencing impaired functionality.

## Methods

### Aims of the study

We aimed to examine the change in work participation and functional status from pre- to post-treatment and 12 months post-treatment among clients at the first 12 PMHC pilot sites, and whether any change differed across gender, age groups, educational level and migration experience. While background variables such as age, gender and education level are commonly investigated, migration experience was included as 1) individuals with such experience are underrepresented among PMHC clients [41], 2) it predicts less improvement in symptoms of anxiety and depression during PMHC treatment [41], and as 3) structural factors related to work participation might affect individuals with migration experience differently than individuals born in Norway. If PMHC is found to give improvement in functional and work status also among individuals with migration experience, this would further encourage working for a more inclusive service.

### Pilot samples and sampling procedure

The pilot sites and sampling procedures are described in detail in previous publications [41, 49]. In short, the first 12 PMHC-pilot sites in Norway were established in 2012–2013 and distributed across several geographical, both urban and rural, areas. Nine of the pilot sites were located in individual municipalities, one through inter-municipal cooperation and two covered boroughs in the Oslo municipality. The population size and demographic profiles of the pilot sites varied substantially [41, 49].

The PMHC teams had on average four full-time equivalents independent of the catchment area population size. As required by the Norwegian Directorate of Health (NDH) guidelines, all teams were multidisciplinary and had at least one clinical psychologist who carried the professional responsibility for the services provided [40]. All therapists had individual treatment responsibilities. All therapists had a minimum of 3 years with relevant higher education, and completed an additional, mandatory one-year training in cognitive behavioural therapy (CBT). Work-focussed CBT was highlighted in the guidelines from NDH as an overarching focus as a means to facilitate return to work and sustainable work participation [40]. Two whole-day seminars during training focussed on how to evaluate work capacity and conduct work-focussed CBT.

The current study was conducted within routine care at the PMHC pilot sites, and clients contacting the services during the inclusion period were invited to participate. All clients first participated in an initial assessment. During this session, information about the content and treatment methodology within PMHC was provided. The therapist also collected the necessary data to decide whether PMHC could be the appropriate treatment, that is the relevance and severity of mental problems, and the available client resources, such as motivation for treatment and social support. Participation in the study was based on opt-in; clients who were suitable for treatment were informed about the study, invited to participate and asked to sign informed consent.

Inclusion criteria were being an inhabitant of the pilot site community, ≥18 years of age, and having mental health needs related to anxiety and/or depression (no formal diagnosis was needed or provided). Therapists received training in recognizing primary characteristics of psychosis, bipolar disorder, personality disorder, severe drug abuse, and suicide risk. Patients with a clear indication, or history, of these problems were generally excluded from PMHC, and referred to their GP or secondary health care services.

### Participants

In total, 2512 clients started treatment at one of the 12 PMHC pilot sites between October 2014 and December 2016. Of these, 1530 (61%) signed informed consent and participated in the study.^1^ The study participation rate varied between 27.7% in Orkdal to 79.3% in Oslo Frogner. In 8 out of 12 pilot sites, the participation rate was over 60%. Of the 1530 participants, 84.6% attended at least two sessions. There were no fixed number of sessions but an intended upper limit of 15 sessions. The median number of scheduled meetings was 6.0 [41].

For the current study, we excluded those 67 years of age and above (*n* = 22) or reporting having retired (*n* = 11), those reporting > 50% disability pension (*n* = 49) and those doing military service (*n* = 2), ending up with a sample of *n* = 1446 participating clients.

### Data collection and measures

The participants were asked to complete questionnaires before first treatment session, before each session during the treatment, at post-treatment, and 12 months after post-treatment. In over 97% of the cases, participants completed the questionnaires electronically. For each participant, therapists (*n* = 68) were asked to complete a questionnaire at post-treatment about the therapy process including degree of work focus in therapy.

Measures were selected to allow for comparison with IAPT (incl. e.g. PHQ, GAD, WSAS) with an adaption to the Norwegian context (e.g. work status questionnaire fitting the Norwegian social security system).

#### Work status

Work status was self-reported and assessed by means of two questions, one multi-response item about current work status, and one multi-response item about sources of income (see Additional file 1 for full questionnaire). Based on these two questions, participants were placed into three categories: 1) *In regular work (part time or full time),* 2) *In work and receiving benefits (*i.e. *fully or graded sickness benefits, graded disability benefits)*, or 3) *Out of work, with or without benefits*. Benefits included sickness benefits, work assessment allowance, disability pension, unemployment benefits, and financial assistance. Students (*n* = 175) were categorized according to their work status. “No work” included unemployment, sheltered employment, disability pension, being a full-time student or homemaker. A similar categorization has been used in another Norwegian treatment study [50] and in previous publications of the PMHC data [41].

#### Functional status

The Work and Social Adjustment Scale (WSAS) [51] was used to measure functional status. WSAS is a simple 5-item self-report measure, which assesses the impact of a person’s mental health difficulties on their ability to function in terms of work or studies, home management, social leisure (activities together with others), private leisure (activities done alone) and personal or family relationships. For each domain the respondent is asked to evaluate degree of impairment during last month on a scale from 0 (not impaired) to 8 (severely impaired). A sum score is calculated, ranging from 0 to 40, where more impairment gives a higher score. The WSAS has also been used for the evaluation of IAPT [39], and there is some evidence that the WSAS in this context displays discriminant validity and has comparable reliability and sensitivity to change as the PHQ-9 and GAD-7 [52].

#### Socio-demographic factors

The following factors were included in descriptive statistics and/or as potential moderators of change in work and functional status over time: Gender, age-group (18-30, 31-49, 50-66), educational level (primary-secondary school, higher education), marital status (having a partner, not having a partner), and migration experience (yes or no; defined as self or both parents being born outside Norway). All variables were self-reported at baseline.

#### Work-focus during treatment

At post-treatment the therapist reported degree of work-focus during treatment on a five-level scale with the response possibilities “very low”, “low”, “some”, “high” and “very high”. A three-level variable was created by merging the first two (very low and low) and latter two (high and very high) categories, keeping the middle category (some) unchanged.

### Ethical considerations

The study was approved by the Regional Ethics Committee for Western Norway (REK-vest no. 2014/597). Informed consent was obtained from all participants, and all were assured that they could withdraw from the study at any time without any consequences for their further treatment.

### Missing data

As previously described, missing questionnaire data were generally low at baseline, but substantially higher at final treatment and 12 months post-treatment [41]. This was also true for proportion of missing data regarding the work status measure (1.0, 45.4, 63.1%) and WSAS (3.8, 46.7, 64.8%), respectively.

Missing data for both work status and WSAS were associated with baseline variables age group and education level with ORs for missing ranging from 1.4–2.1 at final treatment and 2.0–4.1 at 12 months post-treatment for the youngest versus the oldest age group and lowest versus highest educational level, respectively. Missing WSAS data at final treatment were also borderline significantly associated with higher baseline scores of PHQ (OR 1.02), but not with baseline work status, GAD and WSAS score. At 12 months-post-treatment, missing data for work status was additionally weakly associated with male gender (OR 1.3 (95%CI 1.0–1.7) and not having a partner (OR 1.4 (95% CI 1.1–1.8)). Not having a partner was also weakly related to missing data for WSAS at final treatment (OR 1.3 (95% CI 1.0–1.5)) and 12 months (OR 1.5 (95% CI 1.2–1.9)) as well as work status at final treatment (OR 1.4 (95% CI 1.1–1.8)). Missing data for WSAS were additionally related to baseline and final treatment work status (n.s. and 0.7 for the *in work and receiving benefits* group and 1.5 and 1.8 for the *no work* group, compared to the *regular work, no benefits* group). Missing data on work status at 12 months was neither associated with baseline nor final treatment scores of PHQ, GAD and WSAS.

There were moderate to strong correlations between the observed values for both work status and WSAS at baseline and at final treatment (*r* = .65 and *r* = .48) and 12 months post-treatment (*r* = .38 and *r* = .30), as well as between final treatment and 12 months post-treatment (*r* = .47 and *r* = .52). All other relevant baseline variables (gender, age-group, educational level, marital status, migration experience, PHQ-score, and GAD-score) were associated less strongly with the observed values of work status and WSAS at post-treatment and 12-months follow-up (*r* < .3). Note that all associations reported in this section were tested for statistical significance at the *p* < .05 level.

Taken together, the missing analyses gave some indications of data missing at random (MAR). However, apart from the temporal autocorrelations for work status and WSAS, the associations of baseline variables with the observed values of work status and WSAS at post-treatment and 12-months follow-up were relatively weak (*r* < .4). Their impact as auxiliary variables would therefore be low [53].

It’s typical for these kinds of studies that missing data is partly missing not at random (MNAR) as well. Some of the bias introduced by MNAR may, however, be mitigated by including strong correlates of the variables with missing data, which is accomplished in our case by including baseline work status and WSAS in their respective linear mixed models adopting maximum likelihood estimation [53].

### Statistical analyses

Initially, descriptive statistics were conducted, to examine the PMHC samples’ overall work status at baseline, as well as differences (chi squared tests) in work status by central demographic characteristics (gender, age-group, educational level, marital status and migration experience) and degree of work-focus during the course of treatment.

Next, changes in work status and functional status as measured by WSAS from pre- to post- treatment and from post-treatment to 12 months post-treatment were investigated. First, the observed distribution of the work status categories and mean (SD) of the WSAS score at each of the three time points were calculated.

Change in work status was examined by means of multilevel multinomial logit regression models based on maximum likelihood (ML) estimation. Change in WSAS score from baseline to final treatment and 12 months post-treatment were similarly examined using linear mixed-effect regression models, also based on ML-estimation. To account for cluster effects, pilot site was included as a fixed effect in all regression models. ML-estimation yields unbiased estimates under the assumption of MAR and is generally accepted to provide less biased estimates as compared to models that rely on traditional methods for handling missing data (e.g. listwise deletion, last observation carried forward) [53].

To visualize the change in work status and WSAS, predicted margins plots were created based on the mixed-effect models (pilot site; fixed effect, repeated measure on each individual; random effect). We also created table plots to examine in more detail the distribution of transitions between work statuses from baseline to final treatment and from final treatment to 12 months post-treatment. Effect size of change in WSAS score was calculated as estimated mean change/descriptive SD at baseline.

It was also examined whether change over time in work status and WSAS differed across gender, age-group, educational level and migration experience. The educational level variable was dichotomized (primary and secondary school vs. higher education) to improve power and hence increase robustness of the findings. Likelihood ratio tests were performed to determine whether the interaction between time and the respective background variables were statistically significant. The interaction effects were tested without specifying random slopes for the time dummies as a result of identification problems. As this may increase the risk for type-1 errors [54], interaction effects were only considered statistically significant at the *p* < .01 level.

All analyses were conducted using Stata versions 14.0 and 15.0 [55].

## Results

### Baseline description of the working age PMHC sample

Table 1 details the demographic characteristics and work status of the working age sample from the 12 first PMHC pilot sites. As previously reported [41, 49], and also valid for this working-age sample, the PMHC participants included a higher proportion of women, native Norwegians and individuals with higher education compared to the general working-age population in the PMHC catchment areas. More than half of the sample was in the age group 30–49 years old (51.7%), whereas 32.3% were in age group 18–29 and 16.0% in age group 50–66.

**Table 1:** Demographic characteristics of PMHC sample by work status

Variable	Total	Work, without benefits	Work and receive benefits	No work, with or without benefits	
	% (n)	%	%	%	Difference
Total	1446	41.0	38.1	20.9	
Gender					χ^2^ = 21.9, df = 2, *p* < 0.001
Men	25.0 (358)	50.4	28.6	21.0	
Women	75.0 (1073)	37.8	41.4	20.8	
Age group					χ^2^ = 133.8, df = 4, *p* < 0.001
18–29 years	32.3 (462)	49.5	18.9	31.6	
30–49 years	51.7 (741)	40.5	44.2	15.3	
50–66 years	16.0 (229)	25.9	57.9	16.2	
Educational level					χ^2^ = 72.4, df = 4, *p* < 0.001
Primary school	10.0 (144)	25.0	29.2	45.8	
Secondary school	45.0 (643)	42.1	36.3	21.6	
Higher education	45.0 (643)	43.4	42.2	14.4	
Marital status					χ^2^ = 28.0, df = 2, *p* < 0.001
No partner	38.9 (555)	39.3	43.4	17.3	
Having a partner	61.1 (873)	43.8	30.4	25.7	
Migration exp.					χ^2^ = 10.2, df = 2, *p* = 0.006
No	88.6 (1263)	42.3	38.0	19.7	
Yes	11.4 (162)	32.9	37.3	29.8	
Therapist-reported treatment characteristics (data completeness =75.1–78.2%)					
Degree of work focus during therapy					χ^2^ = 42.3, df = 4, *p* < 0.001
Very low-low	22.3 (242)	28.1	13.7	27.2	
Some	47.9 (520)	47.3	47.4	50.0	
High-very high	29.8 (324)	24.6	38.9	22.8	
Collaboration^a^					
No	71.0 (802)	79.8	67.9	58.2	χ^2^ = 36.8, df = 2, *p* < 0.001
GP	16.5 (186)	10.7	21.0	19.3	χ^2^ = 19.4, df = 2, *p* < 0.001
Social insurance	3.6 (41)	0.4	2.8	12.7	χ^2^ = 64.5, df = 2, *p* < 0.001
Work place	0.4 (5)	0.2	0.2	1.4	χ^2^ = 5.5, df = 2, *p* = 0.109

^a^Multiple responses allowed

Looking at work status at baseline, 41.0% reported to be *in regular work,* 38.1% *in work and receiving benefits (fully or graded)*, and 20.9% were *out of work, with or without benefits.* Work status varied by demographic characteristics; As for gender, whereas the proportion *out of work* was similar between men and women, men were more likely to be *in regular work* and women *in work and receiving benefits* (χ^2^ = 21.9, df = 2, *p* < 0.001). Being in regular work was more often reported in the younger age groups, and among those having higher education and natives, than among the older, and those having lower education or migration experience (Table 1). Although the proportion out of regular work was found to be markedly higher than in the general working population, the relative differences in work status by the mentioned sociodemographic characteristics are overall commonly observed in general statistics and previous research [56, 57].

### Treatment characteristics related to work focus

As reported by the therapists, 29.8% of the treatments provided included a high to very high work focus, whereas 47.9% had some work focus and 22.3% little to very little work focus. The degree of work focus was higher in the treatments of those *in work and receiving benefits* at baseline than the other groups (χ2 = 42.3, df = 4, *p* < 0.001) (see Table 1).

In the majority of treatments (71.0%), the therapists reported no collaboration with external instances. Collaboration with the GP was reported for 16.5% and Social Insurance Agency for 3.6% of the cases. Collaboration with the GP and Social Insurance Agency were more common in the groups *not* in regular work than in the group *in* regular work at baseline (Table 1). Collaborated directly with the client’s workplace was rare (0.4% in total).

### Change in work status from baseline to final treatment and 12 months post-treatment

As displayed in Table 2, the observed proportion *in regular work* increased from baseline (40.0%) to final treatment (51.2%), and had further increased at 12 months post-treatment (63.6%). The observed proportion among those *in work and received benefits* decreased over the same time period (38.1, 28.5, 16.1%, respectively), whereas the proportion *out of work* seemed rather stable (20.9, 20.3, 20.3%).

**Table 2:** Observed work status and functional status by WSAS over time

	Baseline	Final treatment	12 months post-treatment
Work status	*n* = 1432	*n* = 789	*n* = 533
In regular work, %	40.0	51.2	63.6
(95% CI)	(38.5–43.6)	(47.7–54.7)	(59.4–67.6)
In work and receive benefits, %	38.1	28.5	16.1
(95%CI)	(35.6–40.6)	(25.5–31.8)	(13.2–19.5)
Out of work, with or without benefits, %	20.9	20.3	20.3
(95%CI)	(18.9–23.1)	(17.6–23.2)	(17.1–23.9)
WSAS^a^	*n* = 1391	*n* = 771	*n* = 509
Mean	18.92	10.61	10.07
(95% CI)	(18.44–19.40)	(9.96–11.27)	(9.19–10.94)

^a^Higher WSAS score signifies more functional impairment (range 0–40)

Results from the multilevel multinomial regression models confirmed a clear and statistically significant improvement in work status, both from baseline to final treatment and from final treatment to 12 months, from *work and receive benefits* to *regular work*. More specifically, setting *regular work* as base, the odds for *work and receive benefits* at final treatment compared to baseline was 0.38 [95% CI 0.29–0.50]. Further on comparing final treatment to 12 months post-treatment, the odds for *work and receive benefits* was again substantially reduced (OR 0.19 [95% CI 0.12–0.32]).

There was on the other hand no statistically significant change in odds for *out of work,* neither when comparing baseline to final treatment (OR 0.73 [95% CI 0.48–1.11]) nor final treatment to 12 months post-treatment (OR 0.76 [95% CI 0.40–1.45]). Also here, *regular work* was set as base.

**Fig. 1:**
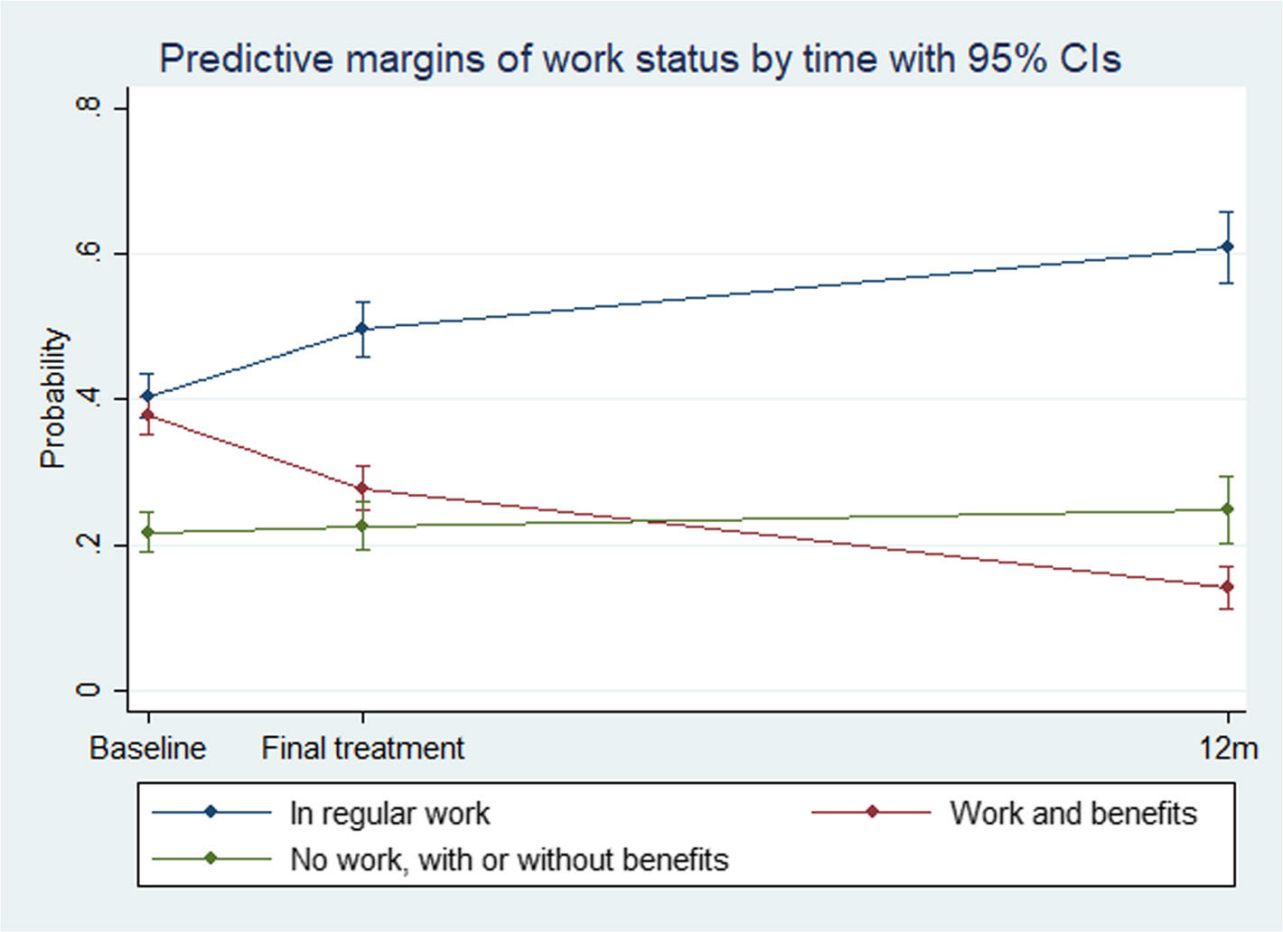
Predicted probability of work status with 95%CI over time based on a multilevel multinomial logit regression model

**Fig. 2:**
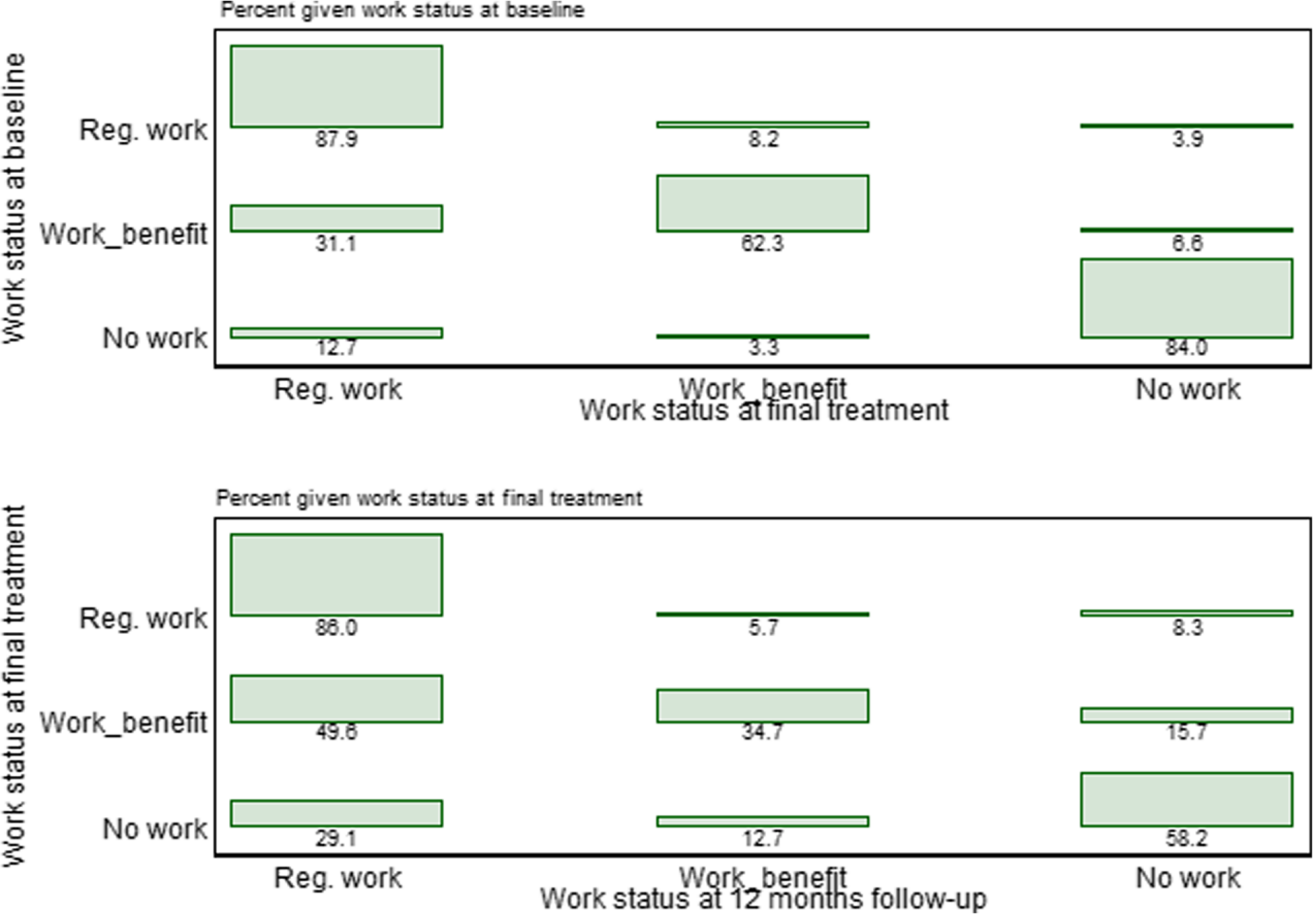
Table plots of transitions in work status from baseline to final treatment (upper figure) and from final treatment to 12 months post-treatment (lower figure)

Figure 1 visualizes the predicted probability for each work status category at baseline, final treatment and 12 months post-treatment. The graph again confirms the increase in probability of *regular work* during follow-up, a corresponding decrease in probability of *work and receive benefits*. The probability of being *out of work*, hardly changed during follow-up. As expected, the ML-based estimated probabilities under the assumption of MAR are different from the observed probabilities presented in Table 1 under the assumption of MCAR. The former indicated somewhat smaller changes at follow-up. In Fig. 2, observed transitions between work statuses from baseline and final treatment, and final treatment and 12 months post-treatment, respectively, are visualized. The figure shows that there are considerably more movement out of the *work and receive benefits* category than the other categories, again indicating that the increase in clients attaining *regular work* mainly is driven by a reduction in those having *work and receive benefits.*

We performed a sensitivity analyses excluding students (*n* = 175), since these as a group has looser work-life attachment, specific health insurance arrangements and “not working” for many equals their normal function as students. The analyses excluding students provided similar results (results not shown).

### Change in functional status by WSAS from baseline to final treatment and 12 months post-treatment

Regarding functional status, the descriptive statistics (Table 2) suggest a substantial improvement in functional status from baseline to final treatment, indicated by a change in mean [95% CI] score of WSAS from 18.92 [18.44–19.40] to 10.61 [9.96–11.27]. At 12 months post-treatment the observed mean WSAS score was still substantially lower than at baseline (10.07 [9.19–10.94]).

**Fig. 3:**
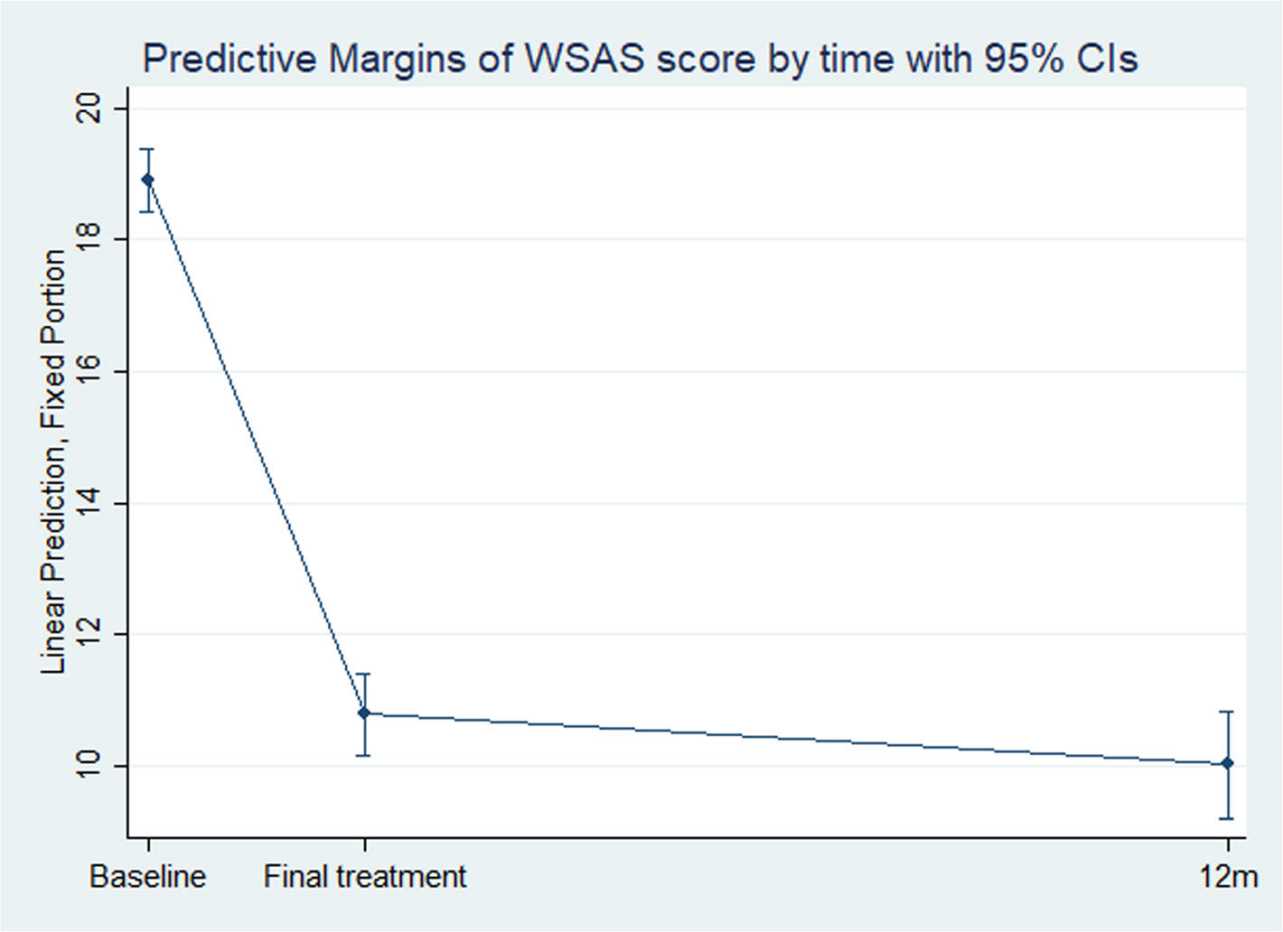
Predicted mean functional status (WSAS) score with 95%CI over time, based on a linear mixed-effect model, adjusted estimates

The linear mixed model confirmed that the change in WSAS score from baseline to final treatment was statistically significant (b = − 8.11 [95% CI, − 8.74 – − 7.48], *p* < 0.001), with a large effect size (ES − 0.89). The observed change from pre- to post-treatment was maintained at 12-month post-treatment, as indicated by a small ES-change (− 0.08) from final treatment to 12 months post-treatment (b = − 0.76 [95% CI, − 1.60 – 0.09], *p* = 0.080). Figure 3 visualizes and confirms the substantial drop in mean predicted WSAS score from baseline to final treatment, and that the change in score levelled off onward to 12 months post-treatment.

Sensitivity analyses, excluding the *n* = 175 students gave the overall same results, only displaying a minor reduction in estimated improvement in WSAS score at final treatment (b = − 7.96 vs b = − 8.11 in total) and 12 months post-treatment (b = − 0.67 vs b = − 0.76 in total).

### Change in work status and functional status by gender, age, education level and migration experience

We did not find evidence for interaction effects between time and any of the variables examined (gender, age, education level and migration experience) for neither work status nor WSAS score. For all these variables the likelihood ratio (LR) tests did not meet the pre-specified requirement of a *p*-value < 0.01.

## Discussion

### Main findings

A clear and substantial increase in regular work participation from pre- to post- treatment was observed among the working-age clients at the 12 first PMHC pilot sites. At 12 months after final treatment the proportion in regular work had further substantially increased. This increase was driven by a corresponding reduction in clients in work and receiving benefits, while there was no statistically significant change over time in the group out of work, with or without benefits. Regarding functional status, a large improvement (effect size = −.89) was observed from pre- to post-treatment, and was maintained 12 months post-treatment. Although our study may have had limited power to detect small differences (effect size ≲.3), our findings did indicate that the change in work status and functional status over time did not differ to a medium-to-large degree by the respective background factors gender, age groups, educational level and migration experience.

### Interpretation

Previous research has found a substantial improvement in symptoms of anxiety and depression during PMHC treatment [41, 58]. The current study shows that PMHC is also associated with substantial improvement in work and functional status from pre- to post-treatment and 12 months post-treatment. Together, this puts weight to the notion of PMHC being a viable alternative for helping adults struggling with mild to moderate anxiety and depression – with regards to symptom levels as well as daily-life functioning and work participation. In light of literature showing that functional impairment may continue also after symptom relief [5, 11, 12], it is highly positive to observe that the strength of the pre-post improvement in functional status (− 0.9) is close to the observed improvement in symptoms of depression (ES-1.1) and anxiety (ES-1.0) in PMHC. Also, knowing that longer absences greatly reduces prospects for return to work [59], observing the transition from receiving benefits to full return to work is encouraging. The observed increase in work participation may have great economic impact, seen both from the individual and societal point of view [60].

However, this study is based on a single-group, pre-post design, and the lack of a control group precludes us from evaluating to what extent the observed improvements are attributable the PMHC treatment. Work status is a multi-causal phenomenon decided by a range of interrelated factors, both proximal such as work ability and more distal such as social security system, the composition of the labour market and unemployment rates [61]. For instance, some of the observed increase in work participation may be driven by system incentives, such as reduced compensation if sick-listed beyond 1 year. Related to this, cross-country differences in benefit systems and how mental disorders are perceived and treated at workplaces [48], as well as cross-study differences in target populations, makes it hard to find suitable benchmark samples. Though the majority of individuals sick-listed with mental disorders in general return to work within a year [62], a range of factors [63], like long or recurrent absences [62, 64], older age [65] and severity and complexity of problems may greatly prolong time to return to work and increase risk of permanent work disability, and may as such limit the effect of PMHC treatment on these outcomes. Thus, we do not know how many would improve their work status also if *not* having received the PMHC treatment. This limitation can only be addressed by comparing outcomes between clients randomized to care in PMHC or to treatment as usual. The on-going randomized controlled trial in two new PMHC sites will be of great interest, addressing the two main limitations of the current study: treatment as usual will be provided to a comparable control group, and problems with missing data at follow-up will be avoided, as work status will be extracted from complete, national registries (ClinicalTrials.gov Identifier: NCT03238872).

Perceived functional status, though situated, may be less affected by structural factors than actual work status. Natural recovery could of course still be an important alternative explanation for the observed improvement. Restricting the analyses to those reporting having problems at least six months prior to treatment (*n* = 1273) did, however, not change effect size of change in functional status. As natural recovery is less likely for individuals with long-lasting problems [66], and as improvement in psychosocial function may lag behind clinical remission [5, 11, 12], at least some of the observed improvement in functional status might be a true benefit of the PMHC treatment. The observed effect sizes of pre-to-post change in WSAS score of Cohens’ d = − 0.89 is somewhat higher than our calculation of findings from IAPT (Cohens’ d: mean pre-post change − 5.07/baseline SD 8.67 = 0.58) [52]. As previously discussed, direct comparisons between IAPT and PMHC should be done with caution [41], as there are differences in clinical population, the treatment models, and in health care system more broadly. Wait-list controls in RCTs in similar settings including functional status outcomes can also serve as benchmark control condition. Few such studies could, however, be identified. Among those identified as having fairly comparable target groups, follow-up times varied considerably. With this in mind, the effect sizes of change in function among the wait-list controls seemed to be in the small to moderate range [67–70], i.e. lower than that observed among the PMHC clients in the current study. Despite the notable limitations to this study, the large and lasting improvement in functional status observed among PMHC clients in this study, can therefore be regarded as promising.

An interesting difference was seen in changes in work status and functional status from final treatment to 12 months post-treatment; while functional status did not change significantly post treatment, similar to previous findings regarding symptoms of depression and anxiety [42], the degree of work participation kept increasing. One the one hand, it cannot be excluded that selective attrition partly could explain the observed pattern. For instance, those participating at 12 months post-treatment might have had better prerequisites (other than measured through WSAS, PHQ or GAD) for returning to work than those dropping out. On the other hand, it could also be argued that those having returned to work might have less time to participate. Another conceivable interpretation is that the observed pattern mirrors the natural course of recovery, were work participation temporally follow symptom and functional improvement [71]. The continuing improvement in work status post treatment could also partly be an effect of the Norwegian sickness insurance scheme, which for instance restrict fully compensated sick-pay to one year. Disregarding of mechanism, the observed “lagged effect” of work participation underscore the importance of having longer-term follow-up in intervention studies with occupational outcomes. Longer follow-up may allow capturing both the whole recovery process, which often takes longer for mental health than many other conditions, and also the degree to which a sustainable work-life attendance is achieved [72].

It should be noted that though one in four of the treatments were reported by therapists to have a high degree of work focus, collaboration with the insurance agency (3.6%) or the client’s workplace (0.4%) was rarely reported. Even for individuals receiving benefits, collaboration with other services was low. In the light of evidence pointing towards service-coordination and workplace accommodations as key components for helping workers with mental health conditions back to work [73], it can be questioned *how* work focus is implemented in the PMHC context. Further studies should examine this in more depth.

The proportion of clients out of work did hardly change from pre- to post-treatment and 12 months post-treatment. At baseline this group was rather heterogeneous, consisting of unemployed (43%), people in sheltered employment (31%), students (27%), homemakers (16%), and people receiving part time (≤ 50%) disability pension (6%) (some had combinations of these statuses). Except for the unemployed, transition to regular work might not be a feasible or relevant treatment goal. That said, in a Norwegian trial of work-focussed CBT with individual job-support (AWaC), it was individuals on long-term benefits at baseline only that showed higher degree of work participation at follow-up [36]. Though the clinical populations in the PMCH and AWaC services are not entirely comparable, one may reason that those on long-term benefits are in particular need of, and may especially profit from, a more comprehensive follow-up regarding work than provided within the current frames of PMHC.

Regarding the findings of no significant interaction effects between time and any of the variables examined (gender, age, education level and migration experience), it is important to consider that the analyses examined difference in *change* and not the absolute differences between the sub-groups. For instance, while there were no difference in change in WSAS score between individuals with and without migration experience, the former group had markedly higher WSAS scores than the latter at all three time points (data not shown). A previous analysis of the PMHC data showed less improvement on symptoms of anxiety and depression among individuals with migration experience compared to those without such experience [41]. It is therefore somewhat surprising, and encouraging, that change in functional status did not differ between these groups. As older age is associated with increased risk of non-return to work [57, 65], it was also unexpected to find no significant differences in change by age groups. The relatively low cut-off at 50 years for oldest age-group and very few clients in the oldest age range in the current study might explain this null-finding.

### Strengths and limitation

Strengths of the current study include the multi-centre and naturalistic design, increasing generalizability of the findings; the inclusion of both functional and work status outcomes, yielding complimentary information about degree of functioning following the PMHC treatment; the use of measures included in previous studies of related samples (WSAS in the IAPT evaluations, work participation measure used in the AWaC studies) facilitating comparability across studies; the relatively long term follow-up of 12 months following final treatment.

The most important limitation is the lack of a control group, which, as discussed above, make it difficult to evaluate to what extent the improvement in work and functional status is attributable to the PMHC treatment. Secondly, the high rates of missing data at post-treatment and 12 months post-treatment, may also have biased the results as discussed above and in the method section. While the degree of bias is difficult to fully ascertain, the use of state-of-the-art methods to deal with missing data is a compensating strength. The relatively strong temporal associations observed for work status and WSAS could partly eliminate the bias introduced by a potential missing not at random (MNAR) situation when using full information maximum likelihood (FIML). Finally, as transitions between work participation and absence can be a dynamic and gradual process [61], pre-post measures might not have fully captured important nuances in the in- or outflow from work.

## Conclusion

Traditionally, clinical trials tend to focus on degree of symptom improvement as its key success criterion [74]. If we indeed intend to sustainably lower the burden of anxiety and depression, we need, however, also to stress the degree to which an intervention succeed in restoring or improving the individuals’ capability to function in their ordinary, daily lives. Symptomatic improvement following the PMCH treatment is previously reported [41, 58]. The current study adds to this knowledge by showing that PMHC treatment is also associated with a substantial and lasting improvement in daily life functioning. Moreover, a considerable amount of clients moved from receiving benefits to work. The degree to which the observed improvements are attributable to the treatment provided need to be confirmed in a trial including a control group, and, ideally, with complete follow-up data from registries. Until then, the present results should be regarded as promising with regard to the effects of PMHC on work participation and functional status.

## Supplementary information

**Additional file 1.** Work situation.

## Data Availability

The datasets analyzed during the current study are not publicly available due to ethical restrictions and personal data protection but are available from the corresponding author on reasonable request.

## Abbreviations

CBT: Cognitive Behavioural Therapy
CI: Confidence Interval
ES: Effect Size
FIML: Full information maximum likelihood
GAD-7: Generalized Anxiety Disorder scale-7
GP: General Practitioner
IAPT: Increased Access to Psychological Therapy
ITT: Intention To Treat sample
LOCF: Last Observation Carried Forward
MAR: Missing At Random
MCAR: Missing Completely At Random
ML: Maximum likelihood
MNAR: Missing Not At Random
OR: Odds Ratio
PHQ-9: Patient Health Questionnaire-9
PMHC: Prompt Mental Health Care
SD: Standard Deviation
WSAS: Work and Social Adjustment Scale

## Footnotes

1 *N* = 1532 was previously reported (Knapstad et al. 2018) but two of these turned out to be test cases and were therefore excluded from the data file.

## References

[1] Lerner D, Henke RM (2008). What does research tell us about depression, job performance, and work productivity?. J Occup Environ Med.

[2] Kroenke K, Spitzer RL, Williams JB, Monahan PO, Löwe B (2007). Anxiety disorders in primary care: prevalence, impairment, comorbidity, and detection. Ann Intern Med.

[3] Bertilsson M, Petersson E-L, Östlund G, Waern M, Hensing G (2013). Capacity to work while depressed and anxious-a phenomenological study. Disabil Rehabil.

[4] Kroenke K, Spitzer RL, Williams JW, Monahan PO, Löwe B (2007). Anxiety disorders in primary care: prevalence, impairment, comorbidity, and detection. Ann Intern Med.

[5] Adler DA, McLaughlin TJ, Rogers WH, Chang H, Lapitsky L, Lerner D (2006). Job performance deficits due to depression. Am J Psychiatry.

[6] Schultz AB, Edington DW (2007). Employee health and Presenteeism: a systematic review. J Occup Rehabil.

[7] Burton WN, Pransky G, Conti DJ, Chen C-Y, Edington DW (2004). The association of medical conditions and presenteeism. J Occup Environ Med.

[8] Moussavi S, Chatterji S, Verdes E, Tandon A, Patel V, Ustun B (2007). Depression, chronic diseases, and decrements in health: results from the world health surveys. Lancet.

[9] Rai D, Skapinakis P, Wiles N, Lewis G, Araya R (2010). Common mental disorders, subthreshold symptoms and disability: longitudinal study. Br J Psychiatry.

[10] Plaisier I, Beekman A, De Graaf R, Smit J, Van Dyck R, Penninx B (2010). Work functioning in persons with depressive and anxiety disorders: the role of specific psychopathological characteristics. J Affect Disord.

[11] Kennedy N, Foy K, Sherazi R, McDonough M, McKeon P (2007). Long-term social functioning after depression treated by psychiatrists: a review. Bipolar Disord.

[12] Saris I, Aghajani M, van der Werff S, van der Wee N, Penninx B (2017). Social functioning in patients with depressive and anxiety disorders. Acta Psychiatr Scand.

[13] Zimmerman M, McGlinchey JB, Posternak MA, Friedman M, Attiullah N, Boerescu D (2006). How should remission from depression be defined? The depressed patient’s perspective. Am J Psychiatry.

[14] Baxter A, Scott K, Vos T, Whiteford H (2013). Global prevalence of anxiety disorders: a systematic review and meta-regression. Psychol Med.

[15] Ferrari A, Somerville A, Baxter A, Norman R, Patten S, Vos T (2013). Global variation in the prevalence and incidence of major depressive disorder: a systematic review of the epidemiological literature. Psychol Med.

[16] Kessler RC, Amminger GP, Aguilar-Gaxiola S, Alonso J, Lee S, Ustun TB (2007). Age of onset of mental disorders: a review of recent literature. Current opinion in psychiatry.

[17] GBD 2013 (2015). Global, regional, and national age–sex specific all-cause and cause-specific mortality for 240 causes of death, 1990–2013: a systematic analysis for the Global Burden of Disease Study 2013. The Lancet.

[18] Knudsen AK, Overland S, Hotopf M, Mykletun A (2012). Lost working years due to mental disorders: an analysis of the Norwegian disability pension registry. PLoS One.

[19] Knudsen AK, Harvey SB, Mykletun A, Overland S (2013). Common mental disorders and long-term sickness absence in a general working population. The Hordaland health study. Acta Psychiatr Scand.

[20] Stansfeld SA, Fuhrer R, Head J (2010). Impact of common mental disorders on sickness absence in an occupational cohort study. Occup Environ Med.

[21] Vaez M, Rylander G, Nygren A, Asberg M, Alexanderson K (2007). Sickness absence and disability pension in a cohort of employees initially on long-term sick leave due to psychiatric disorders in Sweden. Soc Psychiatry Psychiatr Epidemiol.

[22] Cattrell A, Harris EC, Palmer KT, Kim M, Aylward M, Coggon D (2011). Regional trends in awards of incapacity benefit by cause. Occup Med.

[23] Hensing G, Andersson L, Brage S (2006). Increase in sickness absence with psychiatric diagnosis in Norway: a general population-based epidemiologic study of age, gender and regional distribution. BMC Med.

[24] van der Noordt M, IJzelenberg H, Droomers M, Proper KI (2014). Health effects of employment: a systematic review of prospective studies. Occup Environ Med.

[25] McKee-Ryan F, Song Z, Wanberg CR, Kinicki AJ (2005). Psychological and physical well-being during unemployment: a meta analytic study. J Appl Psychol.

[26] Sieurin L, Josephson M, Vingard E (2009). Positive and negative consequences of sick leave for the individual, with special focus on part-time sick leave. Scand J Public Health.

[27] Jahoda M (1981). Work, employment, and unemployment: values, theories, and approaches in social research. Am Psychol.

[28] Torvik FA, Ystrom E, Gustavson K, Rosenström TH, Bramness JG, Gillespie N (2018). Diagnostic and genetic overlap of three common mental disorders in structured interviews and health registries. Acta Psychiatr Scand.

[29] Alonso J, Liu Z, Evans-Lacko S, Sadikova E, Sampson N, Chatterji S (2018). Treatment gap for anxiety disorders is global: results of the world mental health surveys in 21 countries. Depress Anxiety.

[30] Thornicroft G, Chatterji S, Evans-Lacko S, Gruber M, Sampson N, Aguilar-Gaxiola S (2017). Undertreatment of people with major depressive disorder in 21 countries. Br J Psychiatry.

[31] Naidu VV, Giblin E, Burke KM, Madan I (2016). Delivery of cognitive behavioural therapy to workers: a systematic review. Occup Med Oxford.

[32] Joyce S, Modini M, Christensen H, Mykletun A, Bryant R, Mitchell PB (2016). Workplace interventions for common mental disorders: a systematic meta-review. Psychol Med.

[33] Ejeby K, Savitskij R, Öst L-G, Ekbom A, Brandt L, Ramnerö J (2014). Symptom reduction due to psychosocial interventions is not accompanied by a reduction in sick leave: results from a randomized controlled trial in primary care. Scand J Prim Health Care.

[34] Timbie JW, Horvitz-Lennon M, Frank RG, Normand S-LT (2006). A meta-analysis of labor supply effects of interventions for major depressive disorder. Psychiatr Serv.

[35] Nieuwenhuijsen K, Faber B, Verbeek JH, Neumeyer-Gromen A, Hees HL, Verhoeven AC, et al. Interventions to improve return to work in depressed people. Cochrane Database Syst Rev. 2014;(12):CD006237. 10.1002/14651858.CD006237.pub3. 10.1002/14651858.CD006237.pub4 PMC8094165 33052607

[36] Øverland Simon, Grasdal Astrid Louise, Reme Silje Endresen (2018). Long-term effects on income and sickness benefits after work-focused cognitive-behavioural therapy and individual job support: a pragmatic, multicentre, randomised controlled trial. Occupational and Environmental Medicine.

[37] Glasscock DJ, Carstensen O, Dalgaard VL (2018). Recovery from work-related stress: a randomized controlled trial of a stress management intervention in a clinical sample. Int Arch Occup Environ Health.

[38] Salomonsson S, Santoft F, Lindaster E, Ejeby K, Ljtosson B, Ost LG (2017). Cognitive-behavioural therapy and return-to-work intervention for patients on sick leave due to common mental disorders: a randomised controlled trial. Occup Environ Med.

[39] Clark DM, Layard R, Smithies R, Richards DA, Suckling R, Wright B (2009). Improving access to psychological therapy: initial evaluation of two UK demonstration sites. Behav Res Ther.

[40] Helsedirektoratet [Norwegian Directorate of health] (2013). Rask Psykisk Helsehjelp – 12 Pilotkommuner. Veiledende materiell.

[41] Knapstad M, Nordgreen T, Smith ORF (2018). Prompt mental health care, the Norwegian version of IAPT: clinical outcomes and predictors of change in a multicenter cohort study. BMC Psychiatry..

[42] Sæther SMM, Knapstad M, Grey N, Smith ORF (2019). Twelve months post-treatment results from the prompt mental health care (PMHC) program: do improvements last in the Norwegian version of IAPT?. Front Psychol.

[43] Laynard R, Clark D, Knapp M, Mayraz G (2007). Cost-benefit analysis of psychological therapy. Natl Inst Econ Rev.

[44] Layard R, Bell S, Clark D, Knapp M, Meacher M, Priebe S (2006). The depression report: a new deal for depression and anxiety disorders.

[45] Alonso S, Marco JH, Andani J (2017). Reducing the time until psychotherapy initiation reduces sick leave duration in participants diagnosed with anxiety and mood disorders. Clin Psychol Psychother.

[46] Lin EEH, VonKorff MM, Russo JJ, Katon WW, Simon GGE, Unützer JJ (2000). Can depression treatment in primary care reduce disability? A stepped care approach. Arch Fam Med.

[47] Thornicroft G (2018). Improving access to psychological therapies in England. Lancet.

[48] Evans-Lacko S, Knapp M (2014). Importance of social and cultural factors for attitudes, disclosure and time off work for depression: findings from a seven country European study on depression in the workplace. PLoS One.

[49] Smith OR, Alves DE, Knapstad M (2016). Rask Psykisk Helsehjelp: Evaluering av de første 12 pilotene i Norge. [prompt mental health care: evaluation of the first 12 pilot sites in Norway].

[50] Reme SE, Grasdal AL, Løvvik C, Lie SA, Øverland S (2015). Work-focused cognitive–behavioural therapy and individual job support to increase work participation in common mental disorders: a randomised controlled multicentre trial. Occup Environ Med.

[51] Mundt JC, Marks IM, Shear MK, Greist JM (2002). The work and social adjustment scale: a simple measure of impairment in functioning. Br J Psychiatry.

[52] Zahra D, Qureshi A, Henley W, Taylor R, Quinn C, Pooler J (2014). The work and social adjustment scale: reliability, sensitivity and value. Int J Psychiatry Clin Pract.

[53] Enders CK (2010). Applied missing data analysis.

[54] Heisig JP, Schaeffer M (2018). Why you should always include a random slope for the lower-level variable involved in a cross-level interaction.

[55] StataCorp (2017). Stata statistical software: release 15.

[56] Lagerveld SE, Bultmann U, Franche RL, van Dijk FJH, Vlasveld MC, van der Feltz-Cornelis CM (2010). Factors associated with work participation and work functioning in depressed workers: a systematic review. J Occup Rehabil.

[57] de Vries H, Fishta A, Weikert B, Rodriguez Sanchez A, Wegewitz U (2018). Determinants of sickness absence and return to work among employees with common mental disorders: a scoping review. J Occup Rehabil.

[58] Smith OR, Knapstad M, Alves DE, Aarø LE (2017). Initial results of prompt mental health care, the Norwegian version of improving access to psychological therapies. Psychother Psychosom.

[59] Hultin H, Lindholm C, Moller J (2012). Is there an association between long-term sick leave and disability pension and unemployment beyond the effect of health status? - A cohort study. PLoS One.

[60] Chisholm D, Sweeny K, Sheehan P, Rasmussen B, Smit F, Cuijpers P (2016). Scaling-up treatment of depression and anxiety: a global return on investment analysis. Lancet Psychiatry.

[61] Labriola M (2008). Conceptual framework of sickness absence and return to work, focusing on both the individual and the contextual level. Work..

[62] Roelen CA, Norder G, Koopmans PC, van Rhenen W, van der Klink JJ, Bultmann U (2012). Employees sick-listed with mental disorders: who returns to work and when?. J Occup Rehabil.

[63] Blank L, Peters J, Pickvance S, Wilford J, MacDonald E (2008). A systematic review of the factors which predict return to work for people suffering episodes of poor mental health. J Occup Rehabil.

[64] Gjesdal S, Bratberg E (2003). Diagnosis and duration of sickness absence as predictors for disability pension: results from a three-year, multi-register based and prospective study. Scand J Public Health.

[65] Cornelius LR, van der Klink JJL, Groothoff JW, Brouwer S (2011). Prognostic factors of long term disability due to mental disorders: a systematic review. J Occup Rehabil.

[66] Posternak MA, Miller I (2001). Untreated short-term course of major depression: a meta-analysis of outcomes from studies using wait-list control groups. J Affect Disord.

[67] Proudfoot J, Clarke J, Birch M-R, Whitton AE, Parker G, Manicavasagar V (2013). Impact of a mobile phone and web program on symptom and functional outcomes for people with mild-to-moderate depression, anxiety and stress: a randomised controlled trial. BMC Psychiatry.

[68] Richards D, Timulak L, O'Brien E, Hayes C, Vigano N, Sharry J (2015). A randomized controlled trial of an internet-delivered treatment: its potential as a low-intensity community intervention for adults with symptoms of depression. Behav Res Ther.

[69] Kenter RMF, Cuijpers P, Beekman A, van Straten A (2016). Effectiveness of a web-based guided self-help intervention for outpatients with a depressive disorder: short-term results from a randomized controlled trial. J Med Internet Res.

[70] van Straten A, Cuijpers P, Smits N (2008). Effectiveness of a web-based self-help intervention for symptoms of depression, anxiety, and stress: randomized controlled trial. J Med Internet Res.

[71] Sheehan DV, Nakagome K, Asami Y, Pappadopulos EA, Boucher M (2017). Restoring function in major depressive disorder: a systematic review. J Affect Disord.

[72] Holmgren K, Love J, Mardby AC, Hensing G (2014). Remain in work-what work-related factors are associated with sustainable work attendance: a general population-based study of women and men. J Occup Environ Med.

[73] Cullen KL, Irvin E, Collie A, Clay F, Gensby U, Jennings PA (2018). Effectiveness of workplace interventions in return-to-work for musculoskeletal, pain-related and mental health conditions: an update of the evidence and messages for practitioners. J Occup Rehabil.

[74] Papakostas GI, Petersen T, Mahal Y, Mischoulon D, Nierenberg AA, Fava M (2004). Quality of life assessments in major depressive disorder: a review of the literature. Gen Hosp Psychiatry.

