# Supplementary material for: Prompt Mental Health Care (PMHC): work participation and functional status at 12 months post-treatment
Source: BMC Health Serv Res. 2020 Feb 4;20:85. doi: 10.1186/s12913-020-4932-1 (PMC7001227; doi:10.1186/s12913-020-4932-1)
Supplement: Supplementary file 1 — Additional file 1. Work situation. [file 12913_2020_4932_MOESM1_ESM.docx]

**Work situation**

What is your current work situation? Tick ALL alternatives that fit your situation. Please note that a full working week (100% position) consists of 35 hours or more.

☐Full-time worker, not sick-listed

☐Full-time worker, sick-listed

☐Part-time worker, not sick-listed

☐Part-time worker, sick-listed

☐School/education

☐Military service

☐Work-focused program through NAV/municipality

☐Unemployed

☐Disabled

☐Homemaker (i.e., take care of the family home and children)

☐Pensioner/Retired

☐Do not know

What percentage of your current income is based on the following sources of income? Please, only use the categories that fit your situation, so that the various categories add up to 100%.

______% Employment income

______% Sickness allowance

______% Work assessment allowance (AAP)

______% Unemployment benefit

______% Disability benefit

______% Retirement pension

______% Financial assistance (social assistance), individual benefits (NAV)

☐Other, please specify _______________

☐No benefit and/or income

☐Do not know

If you work part-time, what is the percentage of your position?

☐ 0 – 20%

☐ 21 – 40%

☐ 41 – 60%

☐ 61 – 80%

☐ More than 80% but less than 100%

☐ I do not work part time
